# Transcultural adaptation of the children's anxiety questionnaire in Brazil

**DOI:** 10.1002/nop2.794

**Published:** 2021-02-21

**Authors:** Josiane Ramos Garcia Rodrigues, Marla Andréia Garcia de Avila, Milena Temer Jamas, Fernanda Paula Cerantôla Siqueira, Loiane Garcia Daniel, Stefan Nilsson

**Affiliations:** ^1^ Nursing Department Botucatu Medical School ‐ UNESP Botucatu Brazil; ^2^ Nursing Department Marilia Medical School ‐ FAMEMA Marília Brazil; ^3^ School of Architecture Arts and Communication – UNESP Bauru Brazil; ^4^ Institute of Health and Care Sciences, and University of Gothenburg Centre for Person‐Centred Care Sahlgrenska Academy University of Gothenburg Gothenburg Sweden

**Keywords:** anxiety, children, emotional distress, paediatrics, questionnaire

## Abstract

**Aim:**

To describe the transcultural adaptation process of the Children's Anxiety Questionnaire (CAQ) for the Brazilian culture.

**Design:**

This is a methodological study of cross‐cultural adaptation.

**Methods:**

Study conducted in Brazil and Sweden involved the following steps: preparation, translation, synthesis of translations, back‐translation and review, and harmonization of the translations by a committee of 13 healthcare professionals using the content validity index (CVI). Cognitive debriefing, using children between 4–10 years old, was completed by 15 children to determine if the images corresponded with their meanings and 17 children to determine if they could understand the Global CAQ after listening.

**Results:**

Convergences and discrepancies between the original instrument in Swedish, the English version and the Brazilian translation were compared. The process of culturally adapting the CAQ to Brazilian Portuguese was validated, as demonstrated by a satisfactory S‐CVI (0.94) among professionals and an agreement of 95% and above by children.

## 1 INTRODUCTION

Paediatric patients require an extra level of care in the healthcare process. Their care involves more patience, flexibility and restraint on emotions, which are ever changing, as compared to adults. Further, they need to be made to feel safe and receive age‐appropriate information to minimize anxiety and fear, which can impede the delivery of quality health care and have harmful psychological effects in the long term (Lerwick, 2016).

Children who are hospitalized for surgeries or who undergo invasive procedures such as cancer treatment experience various emotions that are often not taken into consideration by healthcare professionals. In general, negative feelings such as fear and anxiety, experienced in response to a threat, can be considered defence reactions. Anxiety is an emotion related to risk assessment that is evoked in situations where the danger is uncertain, either because the context is novel or because the danger stimulus was present in the past (Graeff, 2007). In the hospital setting, when children experience severe anxiety, treatment may be delayed as procedures take longer time to complete, also lowering satisfaction (Lerwick, 2016).

A systematic review of qualitative studies that included patients diagnosed with cancer at or below the age of 18 and reported their perspectives of communicating with clinicians during the treatment of childhood cancers showed that it is important to give voice to children. The findings suggest that even if their choices cannot be honoured, it is important for children to feel “heard” and valued in forming the final decision (Lin et al., 2020).

Children should be made aware of the need for hospitalization and the required procedures to reduce negative feelings such as fear and anxiety. One helpful technique is play therapy, which is used mainly during invasive procedures, to alleviate fear and anxiety (Costa & Morais, 2017). Playing with the child is a simple strategy that boosts the healthcare professional's confidence, comforts the child and facilitates the provision of trauma‐free treatment (Falke et al., 2018).

While participation in decision‐making regarding their treatment can increase children's self‐esteem and adherence to treatment, in reality, such opportunities are limited. Thus, creative communication techniques for use with children can be valuable in clinical practice. In this context, the literature contains descriptions of various strategies and instruments, such as an interactive computer‐based assessment and communication tool (Virkki et al., 2015).

Self‐reports cannot be replaced by assessments by healthcare professionals, since such assessments are likely to measure medical phenomena that do not reflect the subjective experience of the child (Nilsson et al., 2015). There are various scales appropriate for children that allow them to report feelings and symptoms, such as the Children's Fear Scale (McMurtry et al., 2011) to assess fear, the Visual Analog Anxiety Scale (Bringuier et al., 2009) and the Children's Anxiety Questionnaire (CAQ) (Nilsson et al., 2012, 2019) to determine the presence of anxiety.

From a literature review, we identified the absence of appropriate instruments for Brazilian hospital nurses to assess self‐reported anxiety among young children. The CAQ stands out for valuing negative and positive feelings, which makes it useful for the formulation of positive coping strategies. Furthermore, the instrument makes use of images (Nilsson et al., 2019). Thus, there is a need for an instrument that can be used for the evaluation of anxiety in Brazilian children in a manner that takes the features of this population into consideration.

Accordingly, the objective of this study is to describe the process of cross‐cultural adaptation of the CAQ, which facilitates an understanding of both positive and negative feelings in young children, for use in Brazil.

## 2 METHODS

This methodological study of the cross‐cultural adaptation of the CAQ to be used with the Brazilian Portuguese population was carried out between September 2019–June 2020 at two public institutions in Brazil and Sweden.

The following steps were performed according to the literature (Wild et al., 2005): (1) preparation; (2) translation, (3) synthesis of translations, (4) back‐translation and review, (5) harmonization of the translations by the expert committee, (6) cognitive debriefing to test the images of the instrument (Phase 1) and their comprehension and interpretation (Phase 2) by the target population and 7) preparation of the final report.

The CAQ is an instrument created in Sweden, available in English and Swedish, to assess anxiety in children between the ages of five and eight. The authors aimed to develop a questionnaire that would be easy to administer, had concrete psychometric properties and could assess self‐reported anxiety in young children. It is based on the State‐Trait Anxiety Inventory (Spielberger, 1970). The CAQ is a unidimensional scale, and children give their responses based on the four facial expressions, one at a time, and then choose between three steps (i.e. a little, some and a lot). The faces of Happy/Content and Calm/Relaxed are measured as 3 (a little) −2 (some) −1 (a lot), and the faces of Tense/Nervous and Worried/Afraid are measured as 1 (a little) −2 (some) −3 (a lot). This instrument's range is 4 to 12 points, with 4 points signifying no anxiety and 12 points signifying the highest level of anxiety (Nilsson et al., 2012, 2019).

In Step 1, the original Swedish instrument's primary author was involved throughout the translation and adaptation process.

In Step 2, the English instrument was translated by a Brazilian bilingual freelance translator fluent in the original version's language and who was not informed about the objectives of the CAQ. The second translation was performed by a Brazilian nursing professor with a PhD, experienced in validating subjects related to health. She was fully informed about the objectives of the instrument. Two translations (T1 and T2) were obtained and used in Step 2.

In Step 3, both translations were analysed by the researcher, her supervisor and another professor, both with PhD degrees. They identified the most appropriate terms for the questionnaire and integrated them into one version. This process led to the synthesis of the translations (T1‐2).

In Step 4, the synthesis was then back‐translated into the original language of the questionnaire by two other Brazilian independent and bilingual translators, both of whom had no information about the objectives of the questionnaire. Two back‐translations, RT1 and RT2, were reviewed, and no discrepancies were found between the original instrument and the Brazilian version.

In Step 5, the version of the questionnaire obtained in previous stages (T1‐2) was evaluated by a committee of judges. The committee was established after searching the Lattes Platform (http://lattes.cnpq.br/), a virtual curriculum system in Brazil, using the following criteria: (a) knowledge in the area of paediatrics and oncology, (b) good command of the English language and (c) experience in the processes of cultural adaptation and validation of psychometric instruments. Through the search, we identified 61 healthcare professionals who were subsequently requested to perform the evaluations electronically. We considered the following equivalences: (1) semantic: an evaluation based on grammar and vocabulary, (2) idiomatic: expressions that can lose their meaning in another language and can be substituted, (3) conceptual: the evaluation of words with different conceptual meanings in different cultures and (4) cultural: items that must correspond to those experienced in our cultural context. For the equivalence of each item, the experts had to select an option: “equivalent” (4 points), “needs minor revision to be equivalent” (3 points), “needs major revision to be equivalent” (2 points) and “not equivalent” (1 point). We used the content validity index (CVI) to measure the proportion or percentage of the judges' agreement. This method allows the analysis of individual items using the item‐level CVI (I‐CVI), as well as the global instrument S‐CVI (Scale‐Level Content Validity Index) (Polit & Beck, 2006). The calculation of this item‐level content validity index (I‐CVI) was carried out by dividing the number of responses that were considered adequate (3 and 4) by the total number of responses, and the scale's content validity (S‐CVI) was the mean of the I‐CVI. Items with scores of 1 or 2 were then reviewed or eliminated. Final I‐CVI and S‐CVI scores ranged from 0–1. The item was equivalent if the I‐CVI and S‐CVI were greater than or equal to 0.80 (Polit & Beck, 2006). This step resulted in the pre‐test version used in Step 6.

In Step 6, the pre‐test version of the instrument was presented to the target audience in two phases. We included children between 4–10 years old who were selected using convenience sampling. The children, invited by their parents, agreed to participate in the study conducted at the paediatric oncology and haematology units of two public and teaching hospitals in São Paulo. We excluded children diagnosed with neurological diseases and those who could not communicate verbally. Data were collected in a private room by the researcher with experience in paediatric oncology care. In phase 1, children evaluated the instrument to determine if the images used in the questionnaire corresponded with their meanings. They were given two response options: “I agree” and “I do not agree.” We considered a 90% agreement rate (Polit & Beck, 2006).

In phase 2, considering the development of the original CAQ, we adapted to our context and developed Agatha's story to understand if the children had different feelings about Agatha's story and themselves. Children evaluated the instrument to determine the Global CAQ after listening to a researcher telling Agatha`s story (i.e. how do you think Agatha is feeling?) and assessing the child's own emotions (i.e. how are you feeling today?). This story is about a child who has a disease and, accompanied by her mother, is expected to take medication at the hospital. The researcher did not mention that the disease was cancer.

**FIGURE 1:**
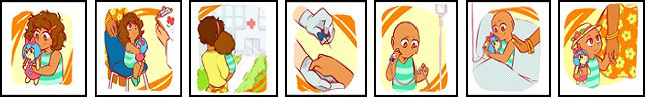
Agatha's story

We expected different responses from the children, with scores lower or higher than Agatha's and we also hoped that some children would identify with the character. If all children had similar scores between themselves and between Agatha's feelings and her own feelings, it would be a negative indication of their understanding of the CAQ (Figure 1).

In Step 7, the final version of the CAQ was developed.

### 2.1 Ethical considerations

This study was approved by the research ethics committee of the institution. All parents provided written informed consent, and they signed for the children after being assured of the confidentiality of their information. All participation was voluntary; participants could withdraw from the study at any time without giving a reason.

## 3 RESULTS

The two versions of translations, T1 and T2, obtained in Step 2, showed differences with regard to “some” and “relaxed,” which one translator considered “reasonable” and “relaxed” and the other “medium” and “quiet.” In Step 2, all terms that differed in T1 and T2 were duly analysed, and after consensus, the summary (T1‐2) was prepared using the terms considered most common in Brazil. In Step 3, the back‐translations (RT1 and RT2) obtained from T1‐2 revealed few differences from the original instrument.

Step 5 consisted of sending the instrument electronically to the 61 selected healthcare professionals identified through the Lattes Platform. The members of the committee of judges were those 13 healthcare professionals who responded within the stipulated 30‐day period. Of the 13 committee members, there were nine women (all nurses) and four men (two doctors, a dentist and a physiotherapist). The mean time after graduation of the judges was 14 (±7.7) years; three of the healthcare professionals had a PhD, two had a post‐doctoral degree, seven had a master's degree, and one of them had specialised in *latu sensu*. The judges worked in different areas: five in paediatric oncology, four in instrument validation, two in paediatric general and two in patient education.

The committee deliberated regarding the term “Medium,” the suggested revision for which was “More or less,” and the term “Little,” which was translated as “A little.” The changes made are described in Table 1.

**TABLE 1:** Comparison of the original version, summary and the changes made to the CAQ after evaluation by the committee of judges

Original version	Summary (T1‐2)	Specialist committee	Children
Happy/Content	Feliz/Alegre	Feliz/Alegre	Feliz/Alegre
Calm/Relaxed	Calmo/Tranquilo	Calmo/Tranquilo	Calmo/Tranquilo
Tense/Nervous	Tenso/Nervoso	Tenso/Nervoso	Tenso/Nervoso
Worried/Afraid	Preocupado/Medo	Preocupado/Medo	Preocupado/Medo
A little	Pouco	Um pouco	Um pouco
Some	Médio	Mais ou menos	Mais ou menos
A lot	Muito	Muito	Muito

The semantic I‐CVI was 1.0, and the idiomatic, conceptual and cultural equivalences were 0.92 each. The S‐CVI was 0.94 (Table 2).

**TABLE 2:** Semantic, idiomatic, conceptual and cultural equivalence

Equivalence	Cultural		Conceptual		Semantic		Idiomatic	
Judge	1 e 2	3 e 4	1 e 2	3 e 4	1 e 2	3 e 4	1 e 2	3 e 4
1		x		x		x		x
2		x		x		x		x
3	**x**		x			x		x
4		x		x		x		x
5		x		x		x		x
6		x		x		x	x	
7		x		x		x		x
8		x		x		x		x
9		x		x		x		x
10		x		x		x		x
11		x		x		x		x
12		x		x		x		x
13		x		x		x		x
I‐CVI	0.92		0.92		1		0.92	

**FIGURE 2:**
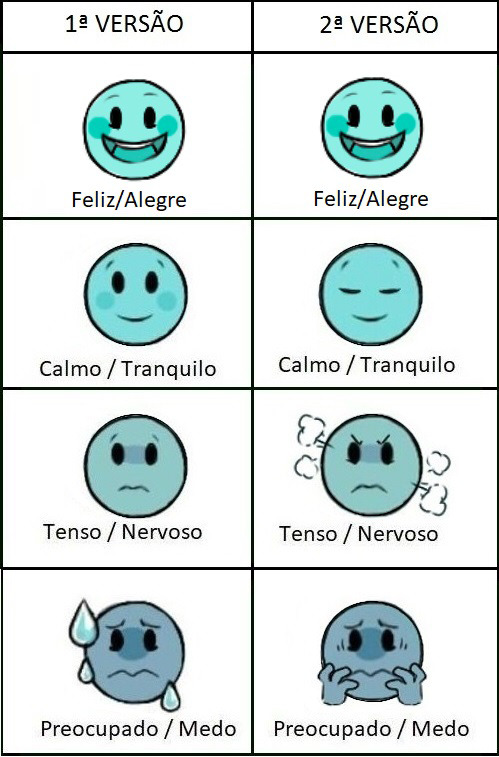
First and second round of Children's Anxiety Questionnaire to the target audience. Botucatu, SP, Brazil, 2020

During the first phase of Step 6, a sample of seven children participated, including five girls, with an average age of six years. Children evaluated the instrument as to whether the images corresponded to the feelings described in them. The response options were “I agree” and “I do not agree,” and the results were as follows: “Happy/Joyful” (100%), “Calm/Tranquil” (85.7%), “Tense/Nervous” (42.8%), “Worried/Fearful” (57.1%) and “A little, More or less, A lot” (100%). This way, images that received an agreement rate below 90% were revised. In the second round of the first phase, the instrument was presented to 15 children (seven from the previous test and eight new children) with a mean age of 5.9 years, 10 of whom were females. The results were as follows: “Happy/Joyful” (100%), “Calm/Tranquil” (93.3%), “Tense/Nervous” (93.3%), “Worried/Fearful” (93.3%) and “More or less,” “A lot” (100%) (Figure 2).

**FIGURE 3:**
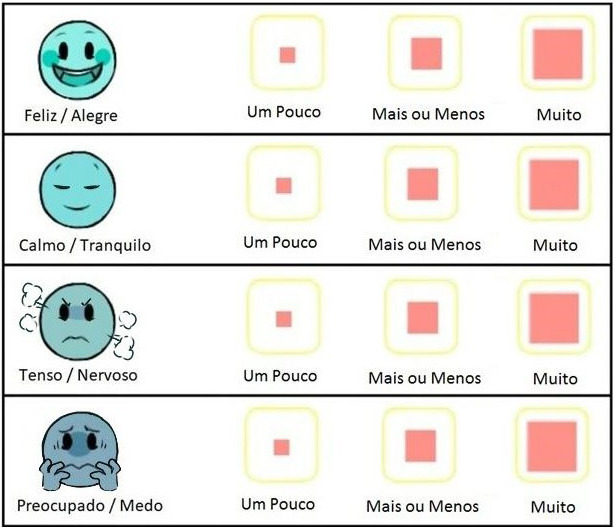
Children's Anxiety Questionnaire‐Brazilian Version

The instrument was titled Children's Anxiety Questionnaire‐Brazilian Version.” (Figure 3).

**FIGURE 4:**
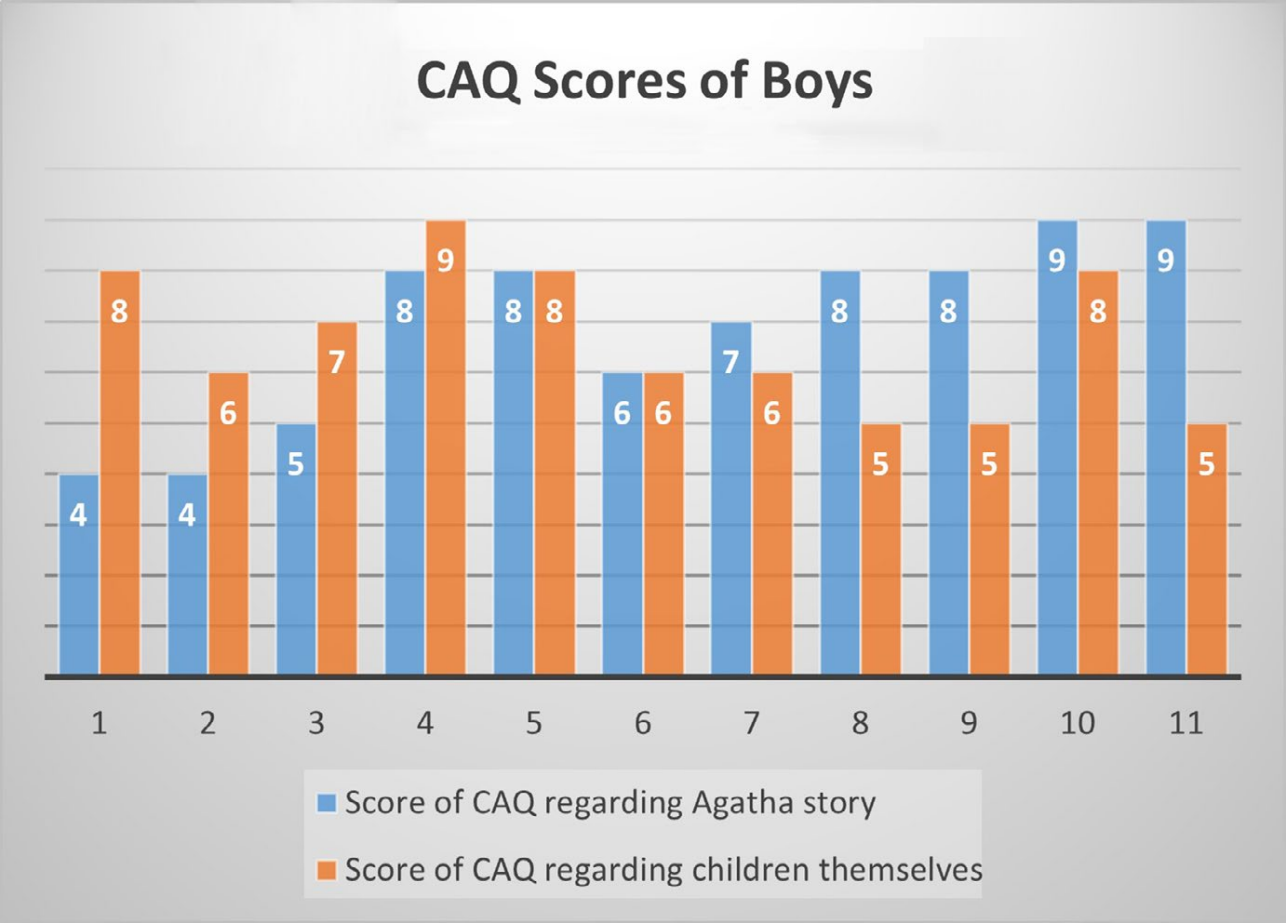
Scores of Boys' Anxiety Questionnaire after Agatha's charge/story and scores of children themselves

**FIGURE 5:**
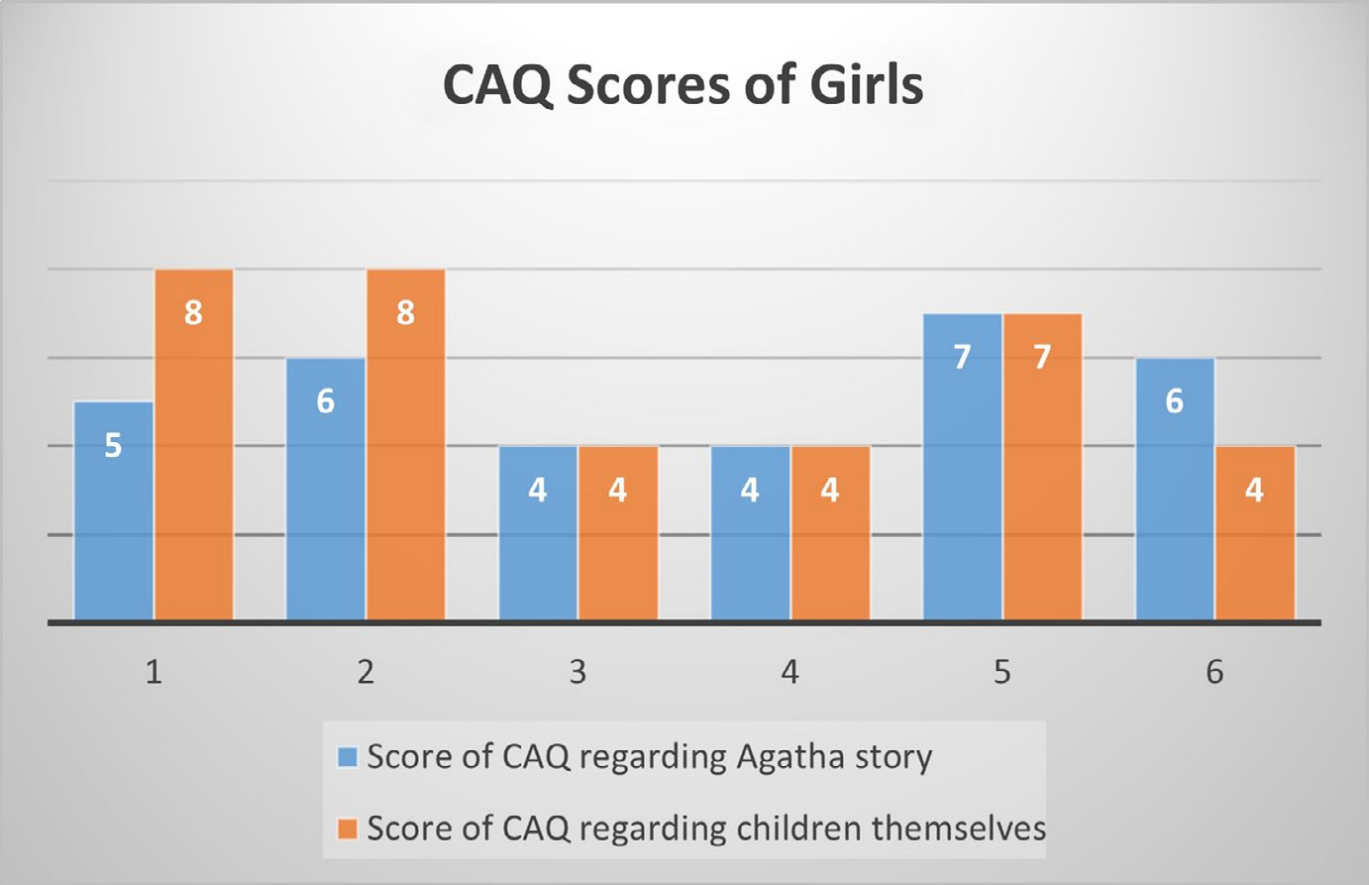
Scores of Girls' Anxiety Questionnaire after Agatha's charge/story and scores of children themselves

In the second phase of Step 6, the instrument was presented to 17 children with a mean age of 7.4 years, 11 of whom were boys. As shown in Figures 4 and 5, boys and girls could use the CAQ to interpret their own emotions as well as Agatha´s emotions in the story. During this step, the instrument was tested on a small group of children in order to evaluate alternative wording and verify the comprehension, interpretation and cultural relevance of the translation. Figures 4 and 5 show that 36.4% (*N* = 4) of boys and 33.3% (*N* = 2) of girls reported lower CAQ scores about Agatha's story than the scores of the children themselves; 18.2% of boys (*N* = 2) and 50% (*N* = 3) of girls reported the same scores in both, and 45.5% (*N* = 5) of boys and 16.7% (*N* = 1) of girls reported higher scores regarding Agatha's story.

During the first phase of Step 7, all the stages to present the Brazilian CAQ were analysed.

## 4 DISCUSSION

This study demonstrates the process of translation and cultural adaptation of the CAQ in Brazil, aimed at developing an instrument that can be incorporated into paediatric nursing to help children voice their emotions. Traditionally, the introduction of instruments created in other cultures or languages was limited to simple translations of the original; however, in this case, a basic translation would have differed too greatly from the original based on the translators’ individual perceptions. Thus, the method adopted in this study was geared towards performing the best possible translation by means of constant verification of equivalences (Schardosim et al., 2014). Moreover, providing instruments adequately adapted to the culture of each country allows the collected data to be reliable and preserves the questionnaire's validity and reliability after the entire adaptation process (Echevarría‐Guanilo et al., 2017).

A qualified group collaborated for the validation of the instrument and evaluation of equivalences, resulting in the replacement of the word “Medium” with “More or less,” which is more commonly used in Brazil. This change brought the scale closer to the culture of the target country without losing its essence. The expertise of different judges resulted in important changes (Epstein et al., 2015).

It is important to note that the Brazilian CAQ makes it possible for children to verbalize both positive and negative feelings. Over the treatment course, children can experience several physical symptoms, and their ability to express their distress is dependent on various factors, such as age, maturity, diagnosis, cognitive status, psychological status, language ability and cultural background. Pictorial support can facilitate children's emotional expression. It is important to emphasize that children also want to be heard and be included in discussions about their care and in smaller decisions (Nilsson et al., 2015).

In this study, children contributed by proposing changes to the images. This step—application to the target audience—is extremely important. Clear images can facilitate use by children who cannot yet read and write. It is important to emphasize that the pre‐test relied on the experiences of each child, taking into account their age and emotions at the time of participation in the study.

Following the process of the original CAQ (Nilsson et al., 2019), we adapted a girl's story to verify if the children understood the CAQ. Children tested the instrument by talking about Agatha's story and their own emotions. Different patterns of response were found, indicating that there was an understanding of the CAQ. However, although our sample is selected by convenient sampling and has more boys, the data suggest that girls tend to identify more with Agatha, and boys reported higher scores when asked about Agatha's feelings. As boys in our culture are usually created to be “stronger” than girls, it is possible that some boys had answered the questionnaire about their perception with lower scores after hearing Agatha's story.

Although increasing numbers of instruments for use in paediatric care are being developed and validated, nurses' lack of knowledge regarding the use of scales for evaluation remains an obstacle (Santos et al., 2017). Subjective situations, such as pain and anxiety in younger children, may be underestimated or devalued by health professionals when they do not use adequate tools for clinical evaluation.

In this sense, the present study holds relevance in paediatric practice because it is concerned with a complex and multidimensional phenomenon—anxiety—present among children, especially when sick and hospitalized (Gomes et al., 2016). Thus, it is opportune to propose that healthcare professionals be trained to ensure adequate assessment of anxiety, appropriate recording and better results regarding its management.

It is also believed that the development of instruments by nurses working in paediatric care settings will enable the performance and qualification of the clinical evaluation as well as documentation of their activities (Marques et al., 2014). In this way, nurses can achieve consensus in the assessment of anxiety and measure it by developmental stage.

Finally, after the completion of all stages of cultural adaptation, the process of validating the CAQ will be initiated in the preoperative and chemotherapy stages to establish its psychometric properties and for subsequent use in new research (Souza et al., 2017).

This study has implications for nursing practice, providing a user‐friendly tool that can facilitate humanized care, improve communication with children and corroborate the paediatric care plan. Identifying anxiety in children is essential for healthcare teams concerning guiding care strategies and planning. In addition, the tool can help monitor changes in behaviour during treatment or follow‐up.

The next step will be to evaluate the psychometric properties of this instrument in a children with cancer diagnosis in a preoperative stage. In addition, due to the pandemic COVID‐19, we used the CAQ to assess the prevalence of anxiety among Brazilian children and its associated factors during social distancing due to COVID‐19 (Garcia de Avila et al., 2020).

Concerning this work, some limitations must be noted in Step 6. The purpose was to evaluate if CAQ assesses anxiety in children between the ages of 4 and 8 years. However, we included children between 4–10 years old in the pre‐test version of the instrument. In addition, Wild et al., 2005, recommends assessing the level of comprehensibility and cognitive equivalence of the translation for a group of five to eight respondents. We applied this sample and did the pre‐test in two different ways, considering that the target is young children. Also, we have presented only one story of a girl, and if we had offered both stories, for a boy and a girl, we may have obtained different results.

## 5 CONCLUSIONS

All phases of the cultural adaptation process were performed, as the literature suggests, and some changes were necessary compared with the original version to attend the Brazilian culture. However, other important issues regarding validity and reliability properties still need to be evaluated.

## CONFLICT OF INTEREST

None.

## AUTHOR CONTRIBUTIONS

Rodrigues JRG: Conceptualization; Data curation; Investigation; Roles/Writing ‐ original draft. Avila MAG: Conceptualization; Investigation; Methodology; Project administration; Resources; Supervision; Visualization; Writing ‐ review & editing. Jamas MT: Conceptualization; Investigation; Methodology; Writing ‐ original draft. Siqueira FPC: Critical revision; Daniel LG: Data curation; Nilsson S: Conceptualization; Methodology; Critical revision; Writing ‐ review & editing.

## Data Availability

The datasets used and/or analysed during the current study are available from the first author on reasonable request.
